# Vaginal Evisceration After Abdominal Hysterectomy: A Case Report

**DOI:** 10.7759/cureus.31191

**Published:** 2022-11-07

**Authors:** Sofia Tsakona, Christos Iavazzo, Alexandros Fotiou, Kalliopi Kokkali, George Vorgias

**Affiliations:** 1 Gynecological Oncology, Metaxa Memorial Cancer Hospital, Piraeus, GRC

**Keywords:** abdominal hysterectomy, complication, bowel, prolapse, vaginal cuff dehiscence

## Abstract

Vaginal cuff dehiscence (VCD) is an extremely rare complication after a hysterectomy, with possible life-threatening consequences. Multiple cases of pelvic organ evisceration through the vaginal cuff have been reported, most frequently precipitated by sexual intercourse. Surgeons should be suspicious of clinical signs of VCD postoperatively, as any intervention should be prompt. Patients at high-risk patients of developing vaginal cuff dehiscence should be advised to refrain from straining exercises for longer periods of time. Herein, we present the case of a 46-year-old with this complication.

## Introduction

Vaginal cuff dehiscence (VCD) is defined as “a full-thickness separation, partial or total, of the anterior and posterior edges of the vaginal cuff.” It is a rare and possibly life-threatening complication after a hysterectomy. The incidence is reported from 0.14% to 4.1% [1-2]. It often requires emergency surgical intervention as a prolapse, and concurrent necrosis, of intraperitoneal content is possible. Vaginal evisceration complicates 35-67% of vaginal cuff dehiscence cases [1-3,4], with the distal ileum as the most frequent eviscerating organ.

It may present as early as three days to months or years after the hysterectomy, typically after sexual intercourse or severe constipation, and may manifest as acute lower abdominal pain, atypical genital bleeding, increased vaginal discharge, or vaginal discomfort. Patients may also present with signs or symptoms of infection or peritonitis [5]. Any of those signs should be followed by an immediate pelvic examination in order to determine the vaginal cuff’s integrity.

The aim of this report is to showcase an incident of vaginal evisceration and compare our approach to the available literature.

## Case presentation

**Figure 1:**
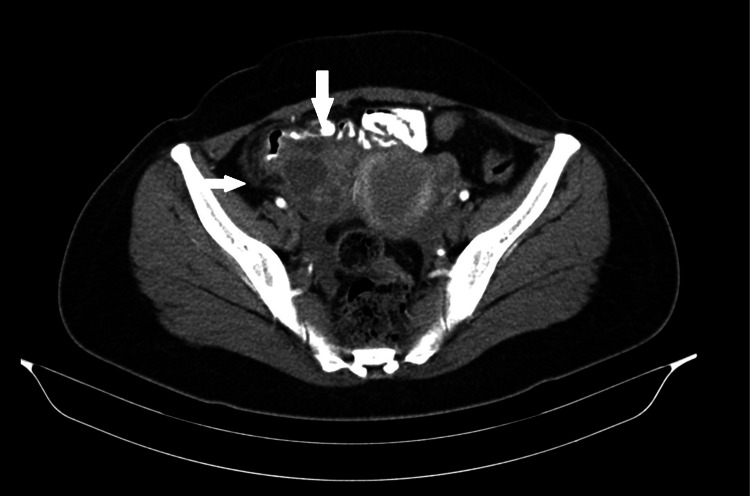
CT scan of the patient revealed a complex cystic mass in the anatomic region of the right ovary

A 46-year-old woman, gravida 2, was admitted to our department with a pelvic mass causing pressure symptoms. She referred a history of left salpingo-oophorectomy for pelvic inflammatory disease three months prior to admission, two caesarian sections, chronic constipation, and smoking (20 pack years). On admission, the computed tomography (CT) scan revealed an ovarian cystic mass so she underwent an abdominal hysterectomy with right salpingo-oophorectomy (Figure 1).

**Figure 2:**
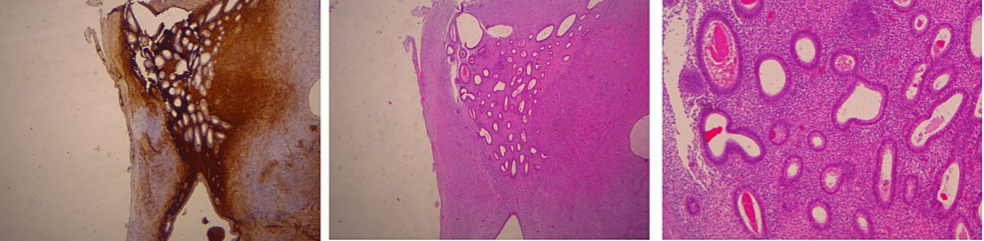
Ovarian endometriosis Endometriotic glands and stroma are visible at the serosa of the ovary. The periglandular stroma is immunoreactive for CD10 as depicted in the first image on the left.

During the laparotomy, the residual pelvic inflammatory disease was identified, in form of abundant peritubal adhesions, as well as endometriotic nodules at the pelvic sidewall. Colpotomy was achieved by using cauterization. The vaginal cuff was closed using two delayed absorbable polyglactin 910 sutures (vicryl) in a continuous one-layer technique. Histopathological findings revealed endometrioma (Figure 2). She was discharged four days later with no notable postoperative complications.

Four weeks post-surgery after straining with defecation and reported intercourse, she experienced abdominal pain with vaginal discharge and was admitted for an emergency laparotomy. After gynecological examination, a full-thickness separation of the vaginal cuff and prolapsed small bowel in the vaginal cavity were identified. Since the viability of the prolapsed tissue could not be ensured, the patient was subjected to abdominal surgery. During the laparotomy, no signs of necrosis or inflammation were identified and the vaginal cuff dehiscence was restored using two delayed absorbable polyglactin 910 sutures (vicryl) in a continuous two-layer technique. Her postoperative recovery was uneventful, with no recurrence of the prolapse in the following six months.

## Discussion

Vaginal cuff dehiscence is an extremely rare, but morbid, complication after a hysterectomy. It usually manifests six to eight weeks after the procedure. In our case, it occurred four weeks postoperatively. The most common symptoms include acute pelvic pain (58-100%), bleeding (23.5-90%), and/or vaginal discharge (55.6%) [1] The addition of abdominal pain with rebound tenderness, with or without fever, suggest peritonitis or bowel ischemia, which has been reported in up to 30% of vaginal cuff eviscerations.

In every case, immediate clinical examination is of paramount importance, to evaluate the extent of the dehiscence as well as the condition of the prolapsed tissue, most commonly the bowel. The gynecological examination of our case revealed small bowel prolapse. If the color of the prolapsed bowel suggests necrosis or the patient appears unstable, immediate laparotomy is in order. However, in most cases, the vaginal approach is sufficient, as the patient appears stable without signs of septic shock or peritoneal involvement. Laparoscopic surgery is gaining ground and should be considered in appropriate cases, as it is minimally invasive and allows peritoneal examination [6,7].

**Figure 3:**
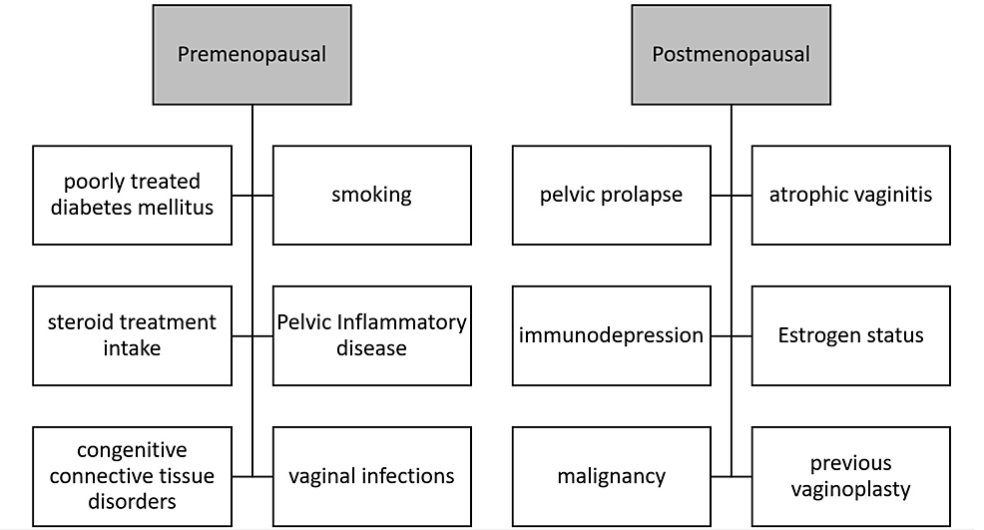
Patient-related high-risk factors for VCD VCD: vaginal cuff dehiscence

Patient risk factors for developing VCD are important in the evaluation of the surgical approach as well as postoperative monitoring. Particularly, instructions regarding the resumption of sexual intercourse are required, especially for patients at high risk for poor wound healing (Figure 3). In premenopausal women, the first postoperative coitus was the most commonly reported precipitating event (8-76%), which occurred six weeks to four months after the hysterectomies [8,9]. It is thus appropriate to consult women to maintain pelvic rest and resume sexual intercourse and tampon use after a minimum of six to eight weeks. In the high-risk group perhaps, this period should extend to 8-12 weeks, giving the vaginal cuff more time to heal [4]. For postmenopausal women, a history of atrophic vaginitis, pelvic prolapse, underlying infection, and/or immunodepression appears to be mostly associated with VCD [8]. It is of paramount importance that any sign of vaginal infection be promptly treated and to prevent constipation in the immediate postoperative period with appropriate diet and/or the use of a stool softener. Independently of the estrogenic status, malignant disease is an independent risk factor for vaginal cuff dehiscence and reported an incidence of 0.8% (9/1,153) after total hysterectomy for malignancy versus 0.2% (4/2,289) when performed for pelvic prolapse [3]. This is in accordance with previous findings, as chemotherapy and/or radiation impact tissue quality and healing. In our case, the risk factors included smoking, chronic constipation, and pelvic inflammation.

Intraoperative factors can also affect vaginal cuff wound healing such as the development of hematoma, infection, and abscess, as well as the chosen type of surgery, the colpotomy technique, the method of cuff closure, and the suture material.

Several studies have confirmed the type of hysterectomy to be a risk factor for VCD. The greater incidence concerns robotic-assisted total laparoscopic hysterectomy (0.40-6.25%), followed by total laparoscopic hysterectomy (0.64-5.83%). laparoscopic-assisted vaginal hysterectomy (0.00-1.68%), total abdominal hysterectomy (0.00-4.76%), and total vaginal hysterectomy (0.00-0.32%), which appears to be the least likely to report cuff dehiscence [1,2,5]. Total abdominal hysterectomy and total vaginal hysterectomy appear to be safer in regards to VDC, however, they lack the distinct advantages of the new techniques, being minimally invasive while maintaining a clear image of the peritoneal cavity. It is unclear whether the higher odds of dehiscence observed after endoscopic techniques are due to a lack of operator expertise, given the later onset of these approaches, or intra-operational factors are linked, such as the application of electrosurgical thermal energy for hemostasis on the vaginal cuff or the theoretical risks due to laparoscopic magnification, which may result in incomplete cuff closure or shallow suture placement.

There are reports associating laparoscopic closure with a higher rate of cuff dehiscences compared to transvaginal sutures (20 of 2,332 0.86% compared with 3 of 1,241: (95% CI 0.53-1.29%)) [1].

The reduction of the incidence of vaginal cuff tears after endoscopic interventions could be achieved by modifications such as the use of bidirectional barbed sutures instead of braided sutures or Endo-Stitch™ (Medtronic plc Dublin, Ireland) which is the most widely used (0% vs. 4.2%, p=0.008) while it doesn’t appear to increase the rate of cuff cellulitis or granulation [10]. Another suggestion is the use of a nonabsorbable suture to prevent spontaneous VCD, however, the second procedure that is required for its removal should be taken into account [11]. Pertaining to the suturing technique, two-layer continuous suturing appears to be the safest method to prevent vaginal dehiscence and postoperative bleeding [4,12].

The use of cauterization on the vaginal cuff inflicts additional tissue damage, which affects negatively tissue healing and is considered a major risk factor for the development of VCD [2]. Therefore it should be avoided during colpotomy and hemostasis, whereas the use of sutures appears to be safer [11,13].

## Conclusions

Vaginal cuff dehiscence is a grave complication that demands immediate intervention. Due to the rarity and heterogeneity of the complication, there is no consensus on how to manage it. The final decision should be tailored to the patient’s clinical needs as well as the surgeon’s expertise. Patients with a higher risk of developing VCD should be carefully monitored and refrain from straining activities for a few weeks longer.
